# Physical constraints on accuracy and persistence during breast cancer cell chemotaxis

**DOI:** 10.1371/journal.pcbi.1006961

**Published:** 2019-04-10

**Authors:** Julien Varennes, Hye-ran Moon, Soutick Saha, Andrew Mugler, Bumsoo Han

**Affiliations:** 1 Department of Physics and Astronomy, Purdue University, West Lafayette, Indiana, United States of America; 2 School of Mechanical Engineering, Purdue University, West Lafayette Indiana, United States of America; 3 Purdue Center for Cancer Research, Purdue University, West Lafayette, Indiana, United States of America; University of Illinois at Urbana-Champaign, UNITED STATES

## Abstract

Directed cell motion in response to an external chemical gradient occurs in many biological phenomena such as wound healing, angiogenesis, and cancer metastasis. Chemotaxis is often characterized by the accuracy, persistence, and speed of cell motion, but whether any of these quantities is physically constrained by the others is poorly understood. Using a combination of theory, simulations, and 3D chemotaxis assays on single metastatic breast cancer cells, we investigate the links among these different aspects of chemotactic performance. In particular, we observe in both experiments and simulations that the chemotactic accuracy, but not the persistence or speed, increases with the gradient strength. We use a random walk model to explain this result and to propose that cells’ chemotactic accuracy and persistence are mutually constrained. Our results suggest that key aspects of chemotactic performance are inherently limited regardless of how favorable the environmental conditions are.

## Author summary

One of the most ubiquitous and important cell behaviors is chemotaxis: the ability to move in the direction of a chemical gradient. Due to its importance, key aspects of chemotaxis have been quantified for a variety of cells, including the accuracy, persistence, and speed of cell motion. However, whether these aspects are mutually constrained is poorly understood. Can a cell be accurate but not persistent, or vice versa? Here we use theory, simulations, and experiments on cancer cells to uncover mutual constraints on the properties of chemotaxis. Our results suggest that accuracy and persistence are mutually constrained.

## Introduction

Chemotaxis plays a crucial role in many biological phenomena such as organism development, immune system targeting, and cancer progression [1–4]. Specifically, recent studies indicate that chemotaxis occurs during metastasis in many different types of cancer [2, 5–9]. At the onset of metastasis, tumor cells invade the surrounding extracellular environment, and oftentimes chemical signals in the environment can direct the migration of invading tumor cells. Several recent experiments have quantified chemotaxis of tumor cells in the presence of different chemoattractants [3] and others have been devoted to the intracellular biochemical processes involved in cell motion [10]. Since the largest cause of death in cancer patients is due to the metastasis, it is important to understand and prevent the directed and chemotactic behavior of invading tumor cells.

Chemotaxis requires sensing, polarization, and motility [11]. A cell’s ability to execute these interrelated aspects of chemotaxis determines its performance. High chemotactic performance can be defined in terms of several properties. Cell motion should be accurate: cells should move in the actual gradient direction, not a different direction. Cell motion should be persistent: cells should not waste effort moving in random directions before ultimately drifting in the correct direction. Cell motion should be fast: cells should arrive at their destination in a timely manner.

**Fig 1:**
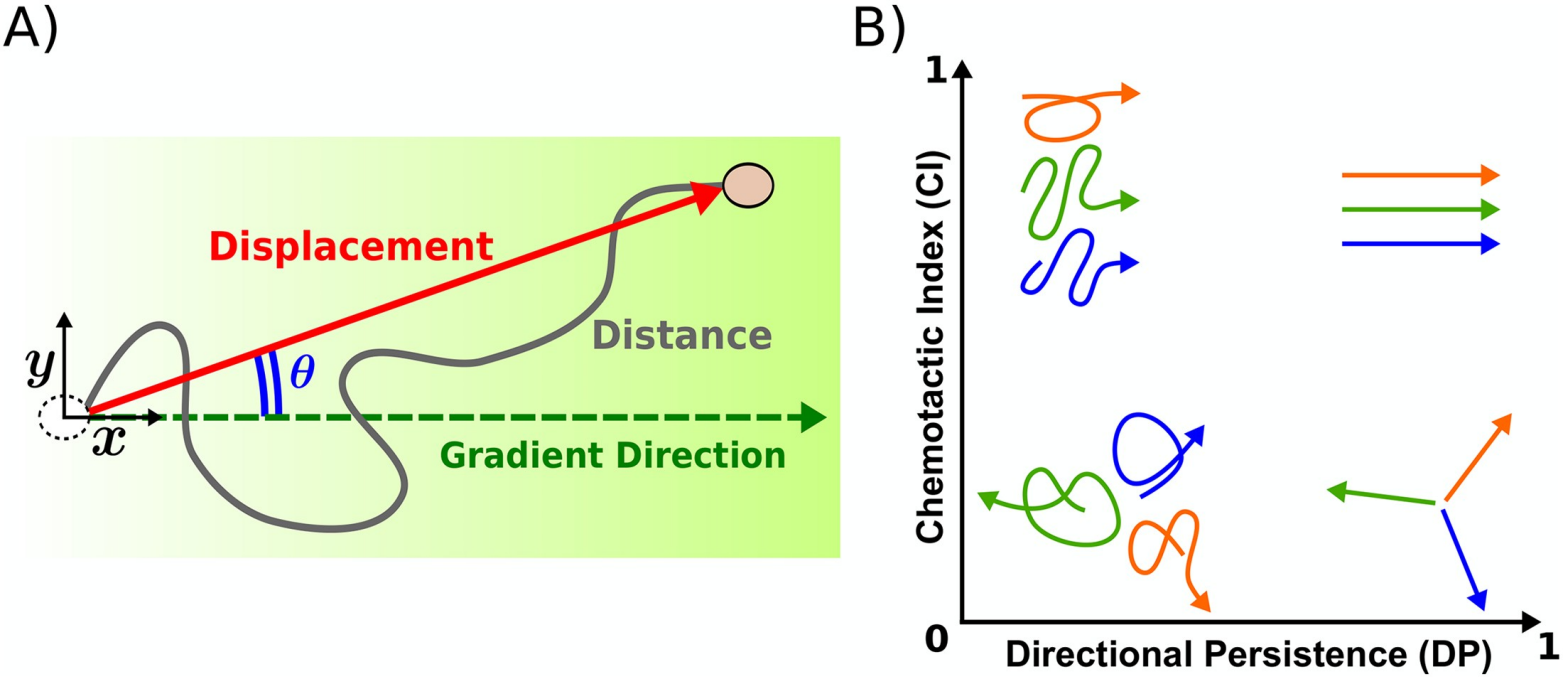
Illustration of chemotaxis. (A) The cell’s displacement makes an angle *θ* with the gradient direction. The chemotactic index (CI) is defined here as the ratio of the displacement in the gradient direction to the total displacement. The directional persistence (DP) is defined here as the ratio of the total displacement to the total distance traveled. (B) High CI values are indicative of cell movement in the gradient direction, whereas high DP values are indicative of straight cell movement in any direction.

Indeed, most studies of chemotaxis use one or more of these measures to quantify chemotactic performance. Accuracy is usually quantified by the so-called chemotactic index (CI), most often defined in terms of the angle made with the gradient direction [12–15] (Fig 1A); although occasionally it is defined in terms of the ratio of distances traveled [16] or number of motile cells [17–19] in the presence vs. absence of the gradient. Directional persistence [10] (DP) is usually quantified by the ratio of the magnitude of the cell’s displacement (in any direction) to the total distance traveled by the cell (Fig 1A; sometimes called the McCutcheon index [20], length ratio [21], or straightness index [22]), although recent work has pointed out advantages of using the directional autocorrelation time [21, 23]. Speed is usually quantified in terms of instantaneous speed along the trajectory or net speed over the entire assay.

However, the relationship among the accuracy, persistence, and speed in chemotaxis, and whether one quantity constrains the others, is not fully understood. Are there cells that are accurate but not very persistent, or persistent but not very accurate (Fig 1B)? If not, is it because such motion is possible but not fit, or is it because some aspect of cell motion fundamentally prohibits this combination of chemotactic properties?

Here we focus on how a cell’s intrinsic migration mechanism as well as properties of the external environment place constraints on its chemotactic performance. The physics of diffusion places inherent limits on a cell’s ability to sense chemical gradients [24]. These limits, along with the cell’s internal information processing and its motility mechanism, determine the accuracy, persistence, and speed of migration. Using a human breast cancer cell line (MDA-MB-231) embedded within a 3D collagen matrix inside a microfluidic device imposing a chemical gradient, we are able to quantify the chemotactic performance of invasive cancer cells in response to various chemical concentration profiles. Results from chemotaxis assays are then compared with simulations and theoretical predictions in order to probe the physical limits of cancer cells to chemotaxis.

## Results

### Quantifying accuracy, persistence, and speed

We measure accuracy using the chemotactic index (CI) [12–15]

$$\begin{array}{c} \text{CI}\equiv \langle \cos\theta \rangle , \end{array}$$ (1)

where *θ* is the angle the cell’s displacement makes with the gradient direction (Fig 1A), and the average is taken over many cell trajectories. CI is bounded between −1 and 1. For chemotaxis in response to an attractant, as in this study, CI generally falls between 0 and 1; whereas in response to a repellent, CI usually falls between −1 and 0. CI = 1 represents perfectly accurate chemotaxis in which cell displacement is parallel to the gradient direction (Fig 1B, top two examples), and CI = 0 indicates that the cells’ migration is unbiased (Fig 1B, bottom two examples). The facts that CI is bounded and dimensionless make it easy to compare different values across different experimental conditions, and get an intuitive picture for the type of cell dynamics it represents.

We measure persistence using the directional persistence (DP), defined as the ratio of the magnitude of the cell’s displacement (in any direction) to the total distance traveled [20–22] (Fig 1A),

$$\begin{array}{c} \text{DP}\equiv \langle \frac{\left|\text{displacement}\right|}{\text{distance}}\rangle . \end{array}$$ (2)

Note that this ratio goes by several names [20–22], and although the name we use here contains the word ‘chemotactic,’ the ratio is in fact independent of the gradient direction. Indeed, DP measures the tendency of a cell to move in a straight line, in any direction. DP is also dimensionless and bounded between 0 and 1, and once again intuitive sense can be made of either limit. If DP = 1, then the cells are moving in perfectly straight lines in any arbitrary direction (Fig 1B, right two examples). In contrast, a low DP is representative of a cell trajectory that starts and ends near the same location on average (Fig 1B, left two examples), with DP → 0 in the limit of an infinitely long non-persistent trajectory.

An alternative measure of persistence is the directional autocorrelation time $\tau_{\text{AC}}=\int_{0}^{\infty }dt^{\prime }\;\langle \cos\left(\theta_{t+t^{\prime }}-\theta_{t}\right)\rangle$, where *t*′ is the time difference between two points in a trajectory, and the average is taken over all starting times *t* [21, 23]. The advantage of the autocorrelation time is that, unlike the DP, it is largely independent of the measurement frequency and total observation time. The disadvantage is that, unlike the DP, it is not dimensionless or bounded. Although we use the DP here, we verify in S1 Fig that the autocorrelation time varies monotonically with the DP for our experimental assay.

We measure speed using the instantaneous speed along the trajectory. That is, we take the distance traveled in the measurement interval Δ*t* (15 minutes in the experiments, see below), divide it by the interval, and average this quantity over all intervals that make up the trajectory.

### Breast cancer cells chemotax up TGF-*β* gradients

We begin by investigating the above properties of chemotaxis in the context of metastasis, specifically the epithelial-mesenchymal transition and subsequent invasion of cancer cells. To this end, we perform experiments using a triple-negative human breast cancer cell line (MDA-MB-231). Invasion of tumor cells *in vivo* is aided by external cues including soluble factors that are thought to form gradients in the tumor microenvironment [2, 5–9]. Among these soluble factors, transforming growth factor-*β* (TGF-*β*) is a key environmental cue for the invasion process [2, 25–28]. Therefore, we use TGF-*β* as the chemoattractant.

**Fig 2:**
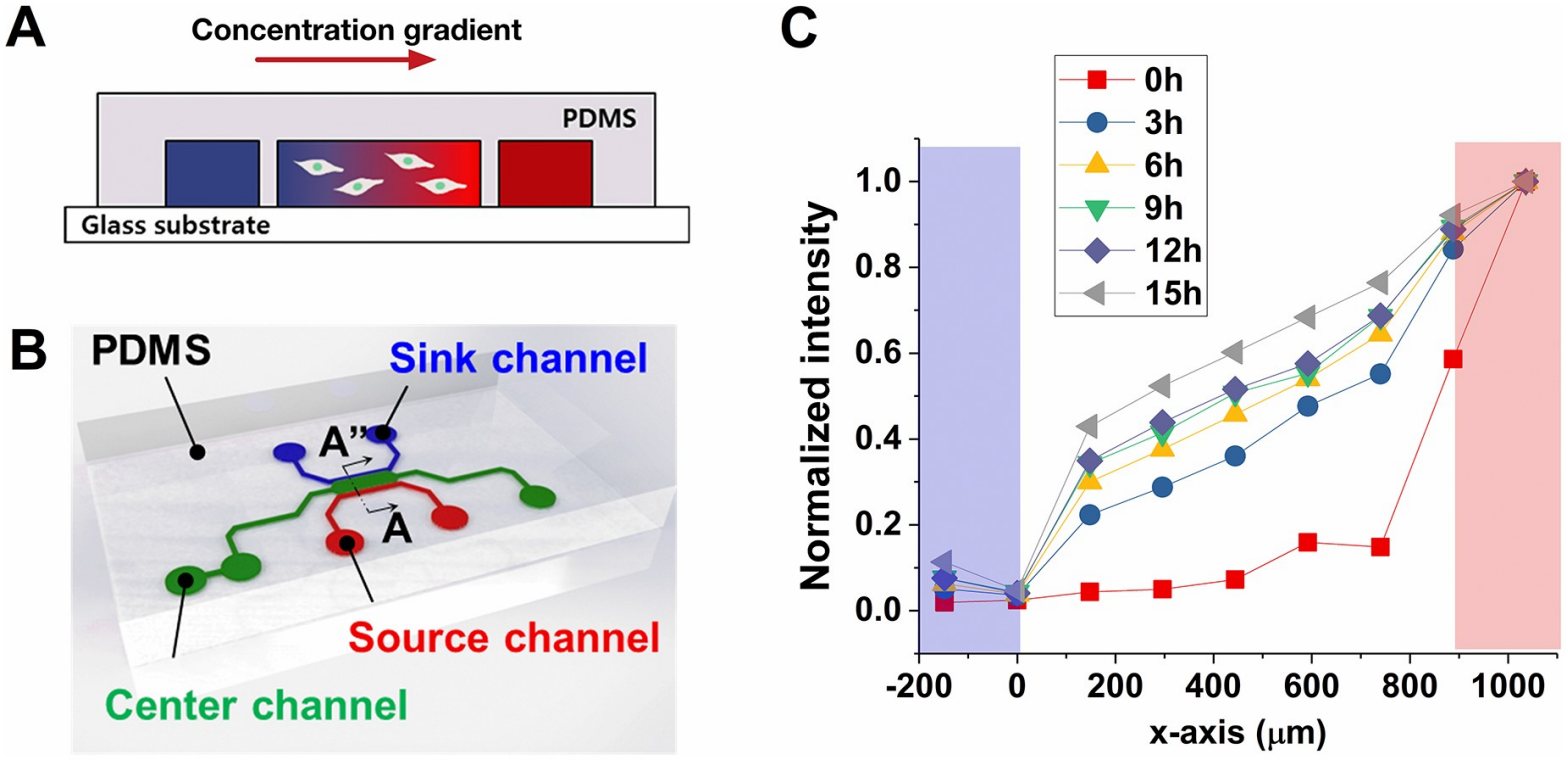
Microfluidic device used as a chemotaxis platform. (A) Cross-sectional view illustrating concentration gradient formed by diffusion. (B) Illustration showing structure of the microfluidic channels. Center channel (green) is filled with type I collagen mixture and MDA-MB-231 mixture, source channel is filled with culture medium containing TGF-*β*, and sink channel is filled with only culture medium. (C) FITC-dextran fluorescence within the center channel. Blue region indicates sink channel while red region indicates source channel.

The *in vivo* tumor microenvironment is highly complex. As a result, *in vitro* platforms have been developed and widely used to investigate the cancer response to a specific cue. In this study, a microfluidic platform is used to expose the TGF-*β* gradient to the cells in 3D culture condition (Fig 2A). The microfluidic device is designed with three different channels, a center, source, and sink channel (Fig 2B). The center channel is filled with a composition of MDA-MB-231 cells and type I collagen while the medium is perfused through the side source and sink channels. TGF-*β* is applied only through the source channel, not the sink channel, and therefore a graded profile develops over time in the center channel by diffusion. Consequently, the MDA-MB-231 cells surrounded by type I collagen are exposed to a chemical gradient of TGF-*β*.

To verify that a graded TGF-*β* profile is generated in the center channel, we utilize 10kDa FITC-dextran, whose hydrodynamic radius (2.3 nm) is similar to that of TGF-*β* (approximately 2.4 nm [29]). The fluorescence intensity is shown in Fig 2C. The profile approaches steady state within 3 hours, is approximately linear, and remains roughly stationary for more than 12 hours. Therefore, we record the MDA-MB-231 cells using time-lapse microscopy every 15 minutes from 3 to 12 hours after imposing the TGF-*β*. See Materials and methods for details.

**Fig 3:**
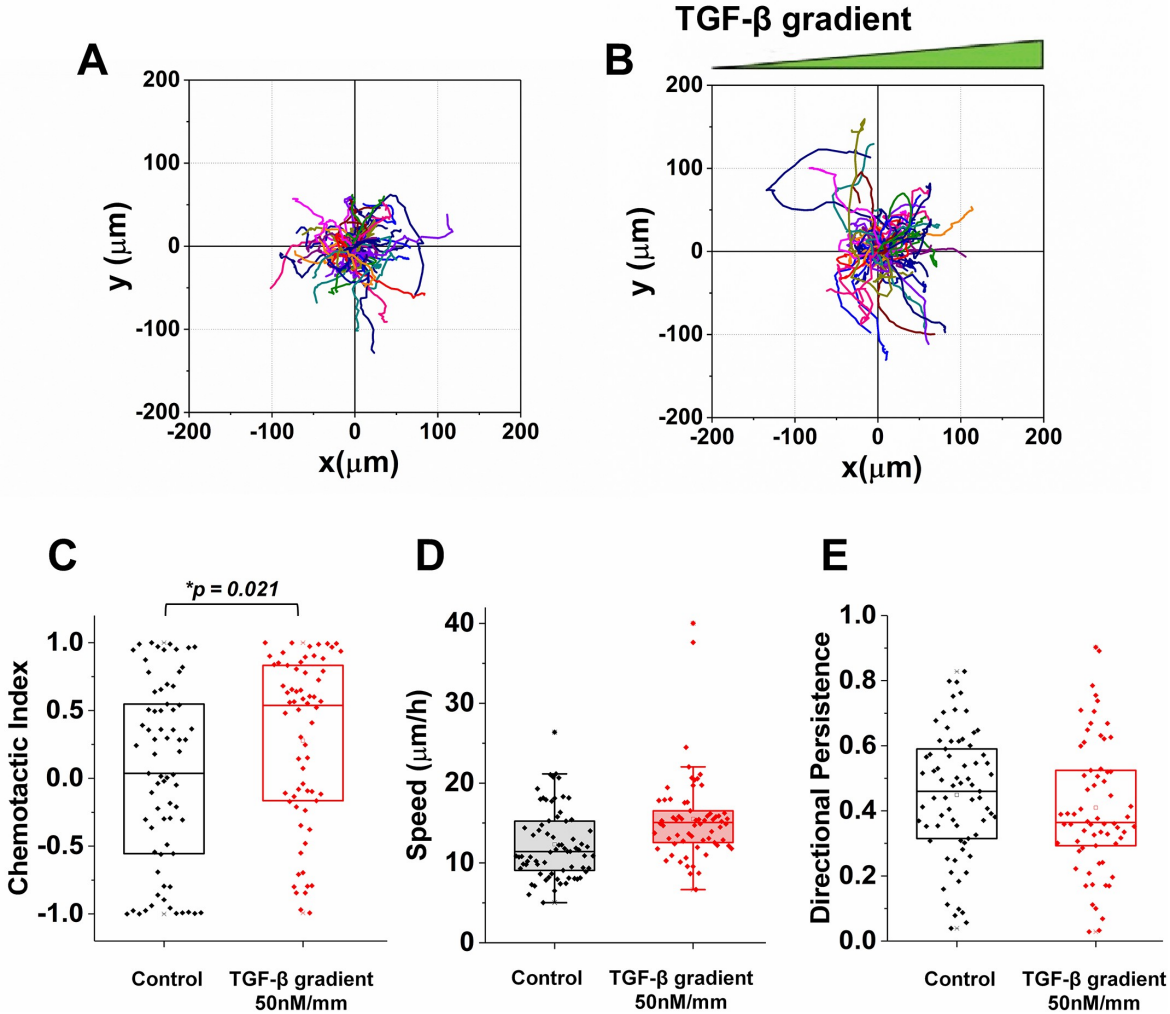
Cell trajectories and chemotaxis metrics. Cell trajectories of (A) control and (B) 50nM/mm TGF-*β* gradient. Distribution of (C) chemotactic index, (D) speed, and (E) directional persistence of each trajectory from both the control (black) and the TGF-*β* gradient (red). Boundary of box plots indicates quadrants with centerline as median. Distributions are statistically compared using Mann-Whitney test.

First, we perform a control experiment with no TGF-*β* to characterize the baseline of the MDA-MB-231 cell migratory behavior. Representative trajectories are shown in Fig 3A, and we see that there is no apparent preferred direction. Indeed, as seen in Fig 3C (black), the CI is centered around zero, indicating no directional bias. Notably, the spread of the CI values is very broad, with many data points falling near the endpoints −1 and 1. This is a generic feature of the CI due to its definition as a cosine: when the distribution of angles *θ* is uniform, the distribution of cos *θ* is skewed toward −1 and 1 because of the cosine’s nonlinear shape. Nonetheless, we see that the median of the CI is very near zero as expected. The speed and DP are shown in Fig 3D and 3E, respectively (black). We see that the DP is significantly above zero, indicating that even in the absence of any chemoattractant, cells exhibit persistent motion. This result is consistent with previous works that showed that cells cultured in 3D tend to have directionally persistent movement unlike those in 2D [10].

Next, we expose cells to a TGF-*β* gradient of *g* = 50 nM/mm. Representative trajectories are shown in Fig 3B, and we see a possible bias in the gradient direction. Indeed, as seen in Fig 3C (red), the CI is centered above zero, indicating a directional bias, and the difference with the control distribution is statistically significant (*p* value < 0.05). We also see in Fig 3D (red) that the speed increases, although we will see below that the increase is relatively small and that the trend is non necessarily monotonic. Finally, we see in Fig 3E (red) that the DP decreases, although the difference with the control is not statistically significant. These results suggest that a TGF-*β* gradient causes a significant increase in directional bias (CI) but not necessarily a significant change in cell speed or persistence (DP).

**Fig 4:**
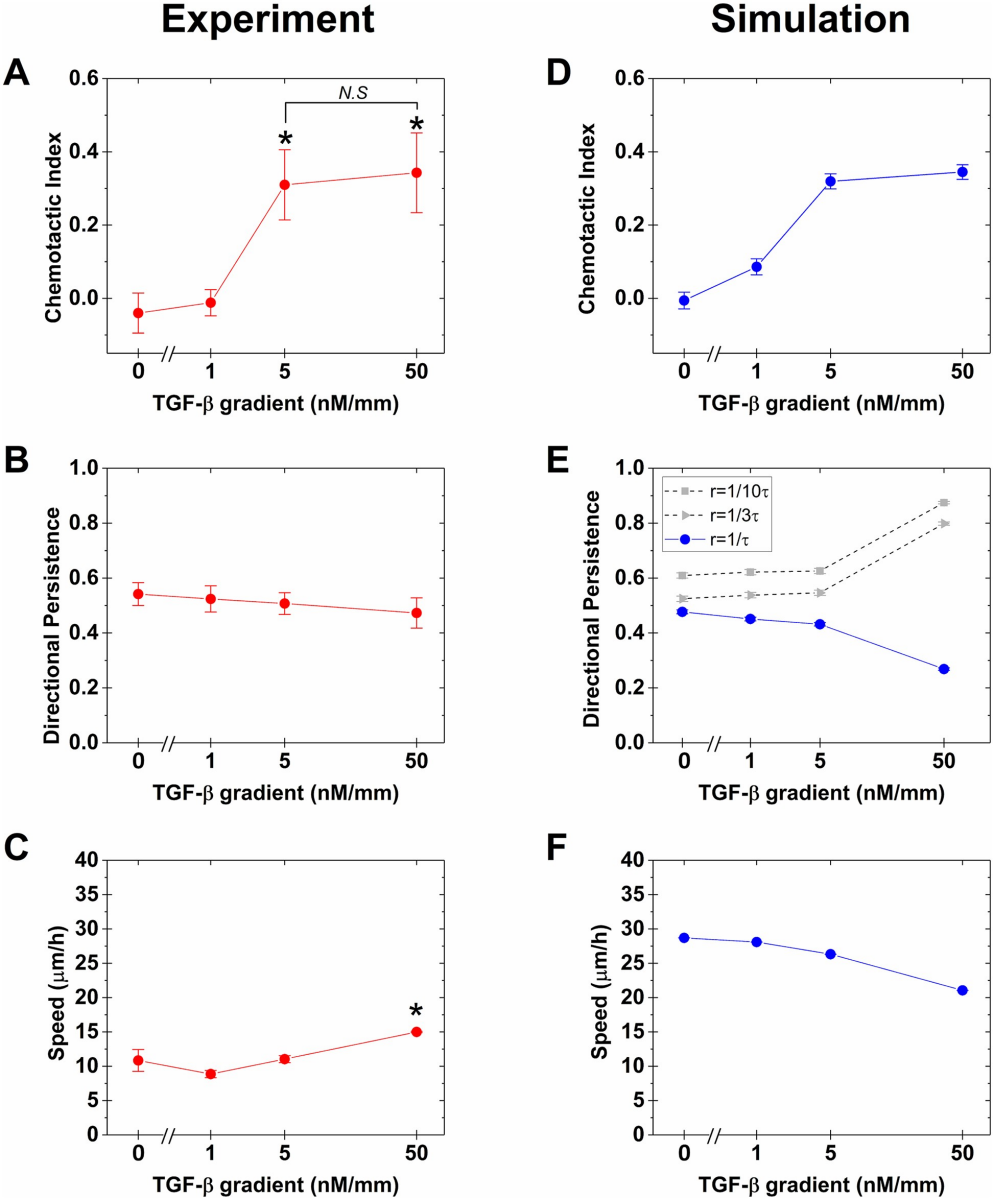
Comparison of experiments with simulations. Experimental (A) chemotactic index, (B) directional persistence, and (C) speed for four different TGF-*β* gradients, *g* = 0, 1, 5, and 50 nM/mm.(red) Data points indicate average and standard error of medians from three different experiments. A, B, and C are plotted with log-scaled TGF-*β* gradient. (D-F) Same for cellular Potts model (CPM) simulations (blue). Error bars are standard error from 1000 trials. Directional persistence from reduced polarization memory decay rate(r) is represented in (E) (gray).

To confirm the trends suggested above, we evaluate the response to four different TGF-*β* gradient strengths, *g* = 0, 1, 5, and 50 nM/mm, in three separate experiments each (Fig 4A–4C; the trajectories for all experiments and *g* values are shown in S2 Fig). We see in Fig 4A that, consistent with Fig 3, the CI is zero for the control and increases with gradient strength *g*. In fact, the CI appears to saturate beyond 5 nM/mm, such that its value at 50 nM/mm is not significantly larger than its value at 5 nM/mm. We also see in Fig 4B, consistent with Fig 3, the DP slightly decreases with the gradient strength although the decrease is roughly within error bars. Finally, we see in Fig 4C that the increase in the speed is small, achieving a statistically significant difference with the control only at the largest gradient strength, and that the trend is not monotonic.

### Minimum detectable gradient is shallow

A striking feature of Fig 4A is that the cells respond to a gradient as shallow as *g* = 5 nM/mm. To put this value in perspective, we estimate both the relative concentration change and the absolute molecule number difference across the cell body [4]. The microfluidic device is about 1 mm in the gradient direction, and therefore a cell in the middle experiences a background concentration of about *c* = 2.5 nM. Assuming the cell is on the order of *a* = 10 *μ*m wide, the change in concentration across its body is *ga* = 0.05 nM, for a relative change of *ga*/*c* = 2%. The number of attractant molecules that would occupy half the cell body is on the order of *ca*^3^ = 1500. Two percent of this is *ga*^4^ = 30, meaning that cells experience about a thirty-molecule difference between their two halves. The same quantities are approximately *ga*/*c* = 1% and 6%, and *ga*^4^ = 60 and 300, for amoebae in cyclic adenosine monophosphate gradients [14] and epithelial cells in epidermal growth factor gradients [30], respectively [4]. This suggests that the response of MDA-MB-231 cells to TGF-*β* gradients is close to the physical detection limit for single cells.

### Simulations suggest sensing and persistence are decoupled

To understand the experimental observation that the CI increases with gradient strength, but the DP and speed do not (Fig 4A–4C), we turn to computer simulations. The cells in the experiments are executing 3D migration through the collagen matrix (as opposed to crawling on top of a 2D substrate). Nevertheless, the imaging is acquired as a 2D projection of the 3D motion. We do not expect this projection to introduce much error into the analysis because the height of the microfluidic device is less than 100 *μ*m, whereas its width in the gradient direction is about 1 mm, and its length is several millimeters. Indeed, from the experimental trajectories (Fig 3) we have estimated that if motility fluctuations in the height direction are equivalent to those in the length direction, then the error in the CI that we make by the fact that we only observe a 2D projection of cell motion is less than 1%. Consequently, for simplicity we use a 2D rather than 3D simulation of chemotaxis of a cell through an extracellular medium.

**Fig 5:**
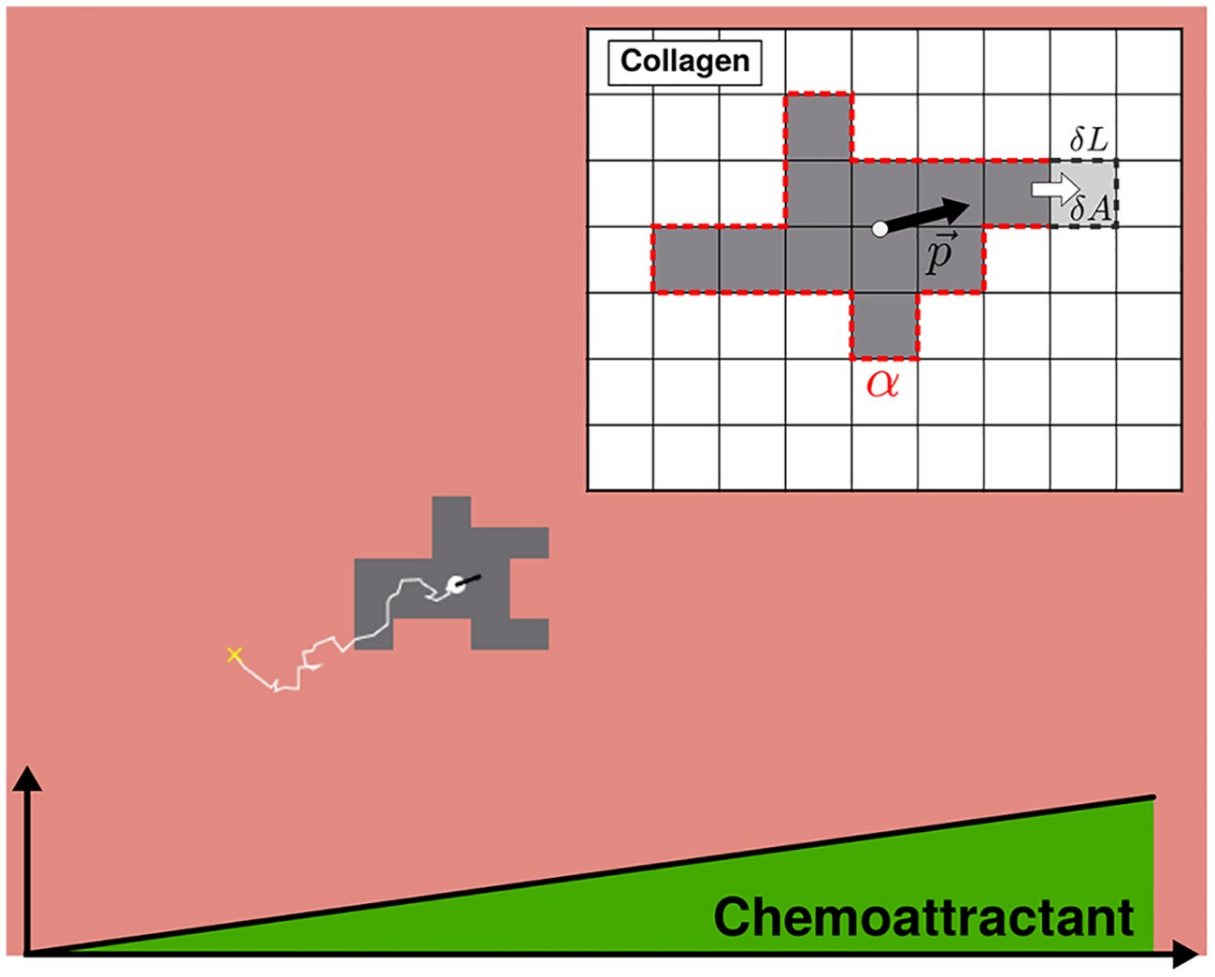
Cellular Potts model (CPM) simulation. Snapshot shows cell (gray) migrating towards increasing chemical concentration over time (white trajectory). Inset: Cell motility occurs through addition and removal of lattice sites. $\vec{p}$, cell polarization vector; *α*, cell-collagen adhesion energy; *δL*, change in perimeter; *δA*, change in area.

Specifically, we use the cellular Potts model (CPM) [31, 32], a lattice-based simulation that has been widely used to model cell migration [33–35] (note that whereas often the CPM is used to model collective migration, here we use it for single-cell migration). In the CPM, a cell is defined as a finite set of simply connected sites on a regular square lattice (Fig 5). The cell adheres to the surrounding collagen with an adhesion energy *α* and has a basal area *A*_0_ from which it can fluctuate at an energetic cost λ. This gives the energy function

$$\begin{array}{c} u=\alpha L+\lambda {\left(A-A_{0}\right)}^{2}, \end{array}$$ (3)

where *L* and *A* are the cell’s perimeter and area, respectively.

Cell motion is a consequence of minimizing the energy *u* subject to thermal noise and a bias term *w* that incorporates the response to the gradient [33]. Specifically, for a lattice with *S* total sites, one update step occurs in a fixed time *τ* and consists of *S* attempts to copy a random site’s label (cell or non-cell) to a randomly chosen neighboring site. Each attempt is accepted with probability

$$\begin{array}{c} P=\{\begin{array}{cc} e^{-(\Delta u-w)} & \;\Delta u-w>0\\ 1 & \;\Delta u-w\leq 0, \end{array} \end{array}$$ (4)

where Δ*u* is the change in energy associated with the attempt. The bias term is defined as

$$\begin{array}{c} w=\Delta \vec{x}\cdot \vec{p}, \end{array}$$ (5)

where $\Delta \vec{x}$ is the change in the cell’s center of mass caused by the attempt, and $\vec{p}$ is the cell’s polarization vector (Fig 5 inset, black arrow), described below. The dot product acts to bias cell motion because movement parallel to the polarization vector results in a more positive *w*, and thus a higher acceptance probability (Eq 4).

The polarization vector is updated every time step *τ* according to

$$\begin{array}{c} \frac{\Delta \vec{p}}{\tau }=r\left(-\vec{p}+\eta \Delta {\hat{x}}_{\tau }+\epsilon \vec{q}\right). \end{array}$$ (6)

The first term in Eq 6 represents exponential decay of $\vec{p}$ at a rate *r*. Thus, *r*^−1^ characterizes the polarization vector’s memory timescale. The second term causes alignment of $\vec{p}$ with $\Delta {\hat{x}}_{\tau }$ according to a strength *η*, where $\Delta {\hat{x}}_{\tau }$ is a unit vector pointing in the direction of the displacement of the center of mass in the previous time step *τ*. Thus, this term promotes persistence because it aligns $\vec{p}$ in the cell’s previous direction of motion. The third term causes alignment of $\vec{p}$ with $\vec{q}$ according to a strength *ϵ*, where $\vec{q}$ contains the gradient sensing information, as defined below. Thus, this term promotes bias of motion in the gradient direction.

The sensing vector $\vec{q}$ is an abstract representation of the cell’s internal gradient sensing network and is defined as

$$\begin{array}{c} \vec{q}=\left\langle \left(n_{i}-\bar{n}\right){\hat{r}}_{i}\right\rangle , \end{array}$$ (7)

where the average is taken over all lattice sites *i* that comprise the cell, and receptor saturation is incorporated as described below. The unit vector ${\hat{r}}_{i}$ points from the cell’s center of mass to site *i*, the integer *n*_*i*_ represents the number of TGF-*β* molecules detected by receptors at site *i*, and $\bar{n}$ is the average of *n*_*i*_ over all sites. The integer *n*_*i*_ is the minimum of two quantities: (i) the number of TGF-*β* receptors at site *i*, which is sampled from a Poisson distribution whose mean is the total receptor number *N* divided by the number of sites; and (ii) the number of TGF-*β* molecules in the vicinity of site *i*, which is sampled from a Poisson distribution whose mean is (*c* + *gx*_*i*_)*ℓ*^3^, where *ℓ* is the lattice spacing, and *x*_*i*_ is the position of site *i* along the gradient direction. Taking the minimum incorporates receptor saturation, since each site cannot detect more attractant molecules than its number of receptors. The subtraction in Eq 7 makes $\vec{q}$ a representation of adaptive gradient sensing: if receptors on one side of the cell detect molecule numbers that are higher than those on the other side, then $\vec{q}$ will point in that direction. Adaptive sensing has been observed in the TGF-*β* pathway [36] in the form of fold-change detection [37] (for shallow gradients, subtraction as in Eq 7 is similar to taking a ratio as in fold-change detection [30]).

The simulation is performed at a fixed background concentration *c* and gradient *g* for a total time *T*. The position of the cell’s center of mass is recorded at time intervals Δ*t*, from which we compute the CI, DP, and speed.

The parameter values used in the simulation are listed in Table 1 and are set in the following way. The values *T* = 9 h, Δ*t* = 15 min, *c* = 2.5 nM, and *g* = 5 nM/mm are taken from the experiments. We estimate *A*_0_ = 400 *μ*m^2^ from the experiments, and we take *ℓ* = 2 *μ*m, such that a cell typically comprises *A*_0_/*ℓ*^2^ = 100 lattice sites. We find that realistic cell motion is sensitive to *α*: when *α* is too small the cell is diffuse and unconnected, whereas when *α* is too large the cell does not move because the cost of perturbing the perimeter is too large. The crossover occurs around *α* ∼ *ℓ*^−1^ as expected, and therefore we set *α* on this order, to *α* = 2 *μ*m^−1^. In contrast, we find that cell motion is not sensitive to λ (apart from λ = 0 for which the cell evaporates), and therefore we set λ = 0.01 *μ*m^−4^ corresponding to typical area fluctuations of λ^−1/2^/*A*_0_ = 2.5%. In order for our Poisson sampling procedure to be valid, the time step *τ* must be much larger than the timescale *ℓ*^2^/*D* for an attractant molecule or receptor to diffuse with coefficient *D* across a lattice site. Taking *D* ∼ 10 *μ*m^2^/s, we find *τ* ≫ 0.4 s. At the other end, we must have *τ* < Δ*t* = 900 s for meaningful data collection. We find that within these bounds, results are not sensitive to *τ*, and therefore we set *τ* on the larger end at *τ* = 100 s to reduce computational run time.

**Table 1:** Table of parameters and values used in cellular Potts model (CPM) simulations. See text for more detailed reasoning behind values.

Parameter	Value	Reason
Total time *T*	9 h	Experiments
Recording interval Δ*t*	15 min	Experiments
Background concentration *c*	2.5 nM	Experiments
Concentration gradient *g*	5 nM/mm	Experiments
Relaxed cell area *A*_0_	400 *μ*m^2^	Experiments
Lattice spacing *ℓ*	2 *μ*m	∼100 sites per cell
Cell-environment contact energy *α*	2 *μ*m^−1^	*α* ∼ *ℓ*^−1^
Area deviation energy λ	0.01 *μ*m^−1^	λ^−1/2^ ≪ *A*_0_
Simulation time *τ*	100 s	*ℓ*^2^/*D* ≪ *τ* < Δ*t*
Total receptor number *N*	10,000	CI saturation
Bias strength *ϵ*	56	Calibrated via CI
Persistence strength *η*	107	Calibrated via DP
Polarization memory decay rate *r*	0.01 s^−1^	*r* ∼ *τ*^−1^

The parameters *N*, *η*, and *ϵ* are calibrated from the experimental data in Fig 4A–4C. Specifically, *N* sets the gradient value above which the CI saturates (see Fig 4A) because if the gradient is large but *N* is small, the cell quickly migrates into a region in which there are more attractant molecules than receptors at all lattice sites, and gradient detection is not possible. We find that *N* = 10,000, which is a reasonable value for the number of TGF-*β* receptors per cell [38, 39], places the saturation level at roughly *g* = 50 nM/mm as in the experiments (Fig 4D). We set *ϵ* = 56 *μ*m^−1^ and *η* = 107 *μ*m^−1^ to calibrate their cognate observables, CI and DP, respectively, to the corresponding experimental values at *g* = 5 nM/mm (Fig 4D and 4E).

The final parameter is the memory timescale of the polarization vector, *r*^−1^. As seen in Fig 4E (gray), we find that the behavior of the DP depends sensitively on this timescale. When *r*^−1^ is large, the DP increases with gradient strength. In contrast, when *r*^−1^ is small (indeed, equal to the smallest timescale in the system, *τ*), the DP does not increase with gradient strength, and in fact slightly decreases (Fig 4E, blue). Because the latter behavior is consistent with the experiments (Fig 4B), we set *r*^−1^ = *τ*. We conclude that the memory timescale of MDA-MB-231 cells is very short when responding to TGF-*β* gradients.

We validate the simulation in two ways, using the speed. First, we find that the magnitude of the speed in the simulations is on the same order as the speed in the experiments (Fig 4C and 4F), i.e., tens of microns per hour. Second, we find that the speed shows little dependence on the gradient strength in both the simulations and the experiments: it slightly increases in Fig 4C and slightly decreases in Fig 4F. Considering that the speed is not calibrated directly in our simulations, these consistencies validate the CPM as a reasonable description of the cell migration in the experiments.

Our finding that the cell’s memory timescale *r*^−1^ takes its minimum value allows for the following interpretation: the parameter *r* couples the persistence term and the sensory term in the CPM (Eq 6). Thus, when the memory timescale *r*^−1^ is long, biased motion must be also persistent and vice versa. In contrast, when the memory timescale *r*^−1^ is short, it is possible for bias to increase without increasing persistence. Therefore, the simulations suggest that the reason that CI but not DP increases with gradient strength in the experiments, is that the drivers of sensory bias and migratory persistence in the cell’s internal network are decoupled from one another.

### Theoretical model reveals performance constraints

Our finding that bias and persistence are decoupled in the simulations allows us to appeal to a much more simplified theoretical model in order to understand and predict global constraints on chemotaxis performance. Specifically, we consider the biased persistence random walk (BPRW) model [40, 41], in which bias and persistence enter as explicitly independent terms controlled by separate parameters. The BPRW has been shown to be sufficient to capture random and directional, but not periodic, behaviors of 3D cell migration [42]. Because we do not observe periodic back-and-forth motion of cells in our experiments, we propose that the BPRW is sufficient to investigate chemotactic constraints here.

As in the simulations, we consider the BPRW model in 2D. In the BPRW model, a cell is idealized as a single point. Its trajectory consists of *M* steps whose lengths are drawn from an exponential distribution. We take *M* = *T*/Δ*t* = 36 as in the experiments. The probability of a step making an angle *θ* with respect to the gradient direction is

$$\begin{array}{c} P\left(\theta |\theta^{\prime }\right)=\underset{\text{bias}}{\underset{︸}{b\cos\theta }}+\underset{\text{persistence}}{\underset{︸}{\frac{e^{p\cos(\theta -\theta^{\prime })}}{2\pi I_{0}\left(p\right)}}}, \end{array}$$ (8)

where *θ*′ is the angle corresponding to the previous step. The first term incorporates the bias, with strength *b*. It is maximal when the step points in the gradient direction (*θ* = 0) and therefore promotes bias in that direction. It integrates to zero over its range (−*π* < *θ* < *π*) because the bias term only reshapes the distribution without adding or subtracting net probability. The second term incorporates the persistence, with strength *p*. It is a von Mises distribution (similar to a Gaussian distribution, but normalized over the finite range −*π* < *θ* < *π*) whose sharpness grows with *p*. It is maximal at the previous angle *θ*′ and therefore promotes persistence. The normalization factor *I*_0_ is the zeroth-order modified Bessel function of the first kind.

**Fig 6:**
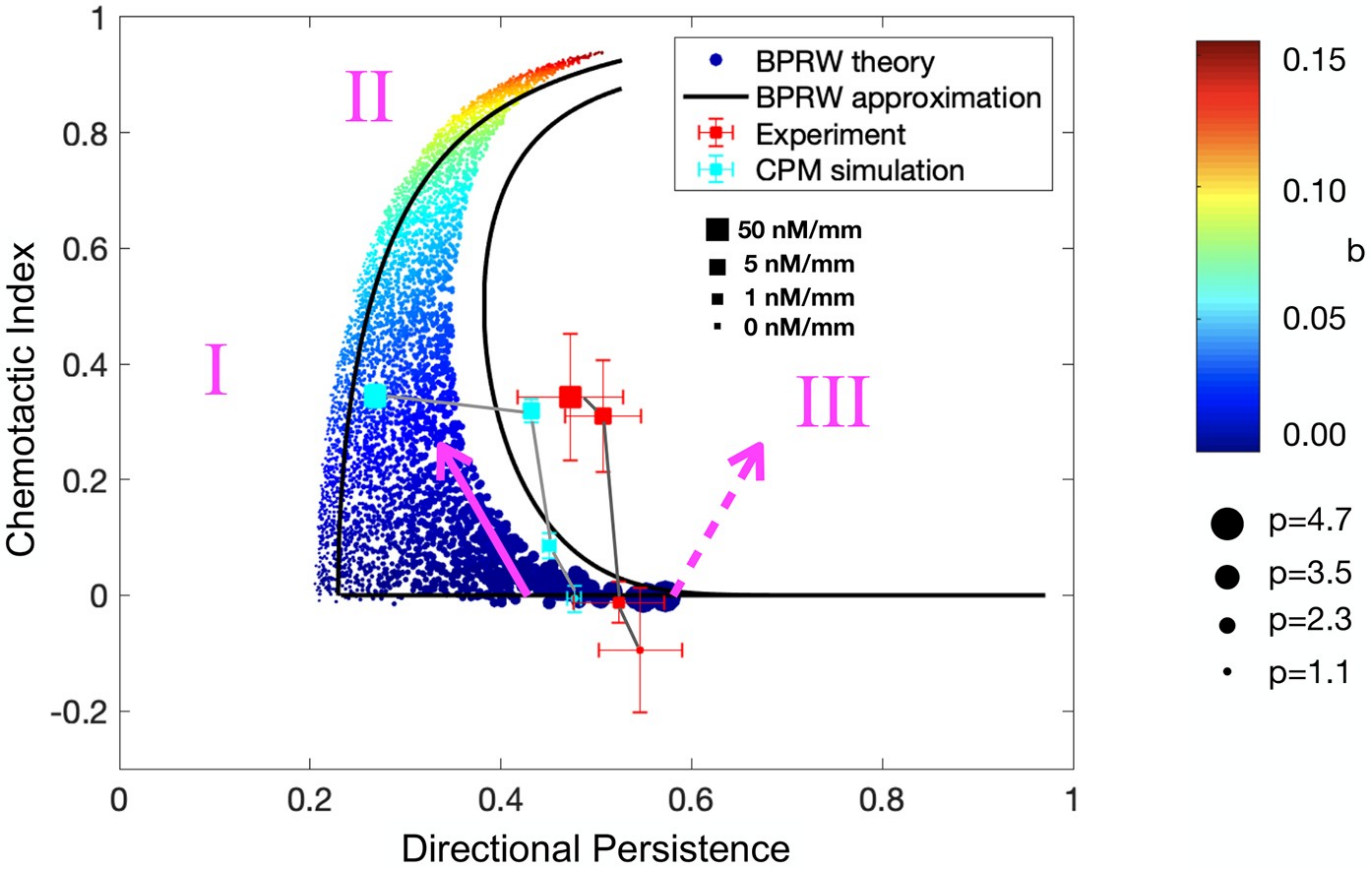
Comparison of theory with experiments and simulations. Colored circles show CI and DP for all values of bias parameter (color) and persistence parameter (size) for biased persistent random walk (BPRW) theory. Black lines show analytic approximations of the bounding curves. Red and cyan squares show experimental and simulation data, respectively, from Fig 4. Magenta numerals and arrows show “forbidden” regions and qualitative trends, respectively, discussed in text.

The requirement that *P*(*θ*|*θ*′) be non-negative over the entire range of *θ* mutually constrains *b* and *p*. However, apart from this constraint, *b* and *p* can take any positive value. We sample many pairs of *b* and *p*, reject those that violate the constraint, and compute the CI and DP from a trajectory generated by each remaining pair. The results are shown in Fig 6 (colored circles). We see in Fig 6 that the BPRW model exists in a highly restricted ‘crescent’ shape within CI–DP space. As expected, the CI increases with the bias parameter *b* (color of circles, from blue to red). The top corner corresponds to maximal bias and no persistence; indeed, when *p* = 0 the persistence term in Eq 8 reduces to (2*π*)^−1^, and non-negativity requires *b* < (2*π*)^−1^ ≈ 0.16, which is consistent with the upper limit of the color bar. Also as expected, the DP increases with the persistence parameter *p* (size of circles, from small to large), although only in the lower portion where the CI is low.

The crescent shape of the allowed CI and DP values in Fig 6 can be understood quantitatively because several moments of the BPRW are known analytically [41]. Specifically, the mean squared displacement and the mean displacement in the gradient direction are, in units of the mean step length,

$$\begin{array}{cc} \left\langle r^{2}\right\rangle & =\frac{1}{{\left(1-\psi \right)}^{2}}[z^{2}{\tilde{M}}^{2}+2(1-2z^{2}-z^{2}e^{-\tilde{M}})\tilde{M}\\ & \;+2(2z^{2}-1)(1-e^{-\tilde{M}})+2z^{2}(1-e^{-\tilde{M}}{)}^{2}], \end{array}$$ (9)

$$\begin{array}{cc} \left\langle x\right\rangle & =\frac{z}{1-\psi }(\tilde{M}-1+e^{-\tilde{M}}), \end{array}$$ (10)

respectively, where $\tilde{M}=M\left(1-\psi \right)$ and *z* = *χ*/(1 − *ψ*), with $\chi =\int_{-\pi }^{\pi }d\theta \;b\cos^{2}\theta =\pi b$ and $\psi =\int_{-\pi }^{\pi }d\phi \;{\left[2\pi I_{0}\left(p\right)\right]}^{-1}e^{p\cos\phi }\cos\phi =I_{1}\left(p\right)/I_{0}\left(p\right)$. We approximate the CI and DP in terms of these moments,

$$\begin{array}{c} \text{CI}=\langle \frac{x}{r}\rangle \approx \frac{\left\langle x\right\rangle }{\left\langle r\right\rangle }\approx \frac{\left\langle x\right\rangle }{\sqrt{\left\langle r^{2}\right\rangle }}, \end{array}$$ (11)

$$\begin{array}{c} \text{DP}=\frac{\left\langle r\right\rangle }{M}\approx \frac{\sqrt{\left\langle r^{2}\right\rangle }}{M}, \end{array}$$ (12)

and evaluate these expressions in specific limits to approximate the edges of the shape. In the limit *b* = 0, Eq 11 reduces to CI = 0 (bottom black line in Fig 6). In the limit *p* = 0, Eqs 11 and 12 are functions of only *b* and *M*, and *b* can be eliminated to yield $\text{DP}={\left[1+M\left(1-{\text{CI}}^{2}\right)/2\right]}^{-1/2}$ (left black line in Fig 6), where we have used the approximation *M* ≫ 1 (see Materials and methods). Note here that when CI = 0 we have DP ≈ (*M*/2)^−1/2^ for large *M*, which makes sense because for a simple random walk (*p* = *b* = 0) the displacement goes like *M*^1/2^ while the distance goes like *M*, such that DP ∼ *M*^−1/2^. Finally, the right edge corresponds to the maximal value of *p* for a given *b*, for which we compute the approximation curve parametrically (right black line in Fig 6; see Materials and methods). We see in Fig 6 that these approximate expressions slightly underestimate the CI and overestimate the DP, but otherwise capture the crescent shape well. The under- and overestimation are due to the approximation $\langle r\rangle \approx \sqrt{\langle r^{2}\rangle }$ in Eqs 11 and 12: because $\sigma_{r}^{2}=\langle r^{2}\rangle -{\langle r\rangle }^{2}\geq 0$ for any statistical quantity, we have $\sqrt{\langle r^{2}\rangle }\geq \langle r\rangle$, making Eq 11 an underestimate and Eq 12 an overestimate.

The crescent shape can also be understood intuitively. First, we see that the DP cannot be smaller than a minimum value (region I in Fig 6). This is because the trajectory length *M* is finite, and as discussed above, the DP only vanishes for infinitely long trajectories. If *M* were to increase, the crescent would extend further toward DP = 0. Second, we see that the top of the crescent bends away from the CI →1, DP →0 corner (region II in Fig 6). In other words, it is not possible to have high bias without any persistence. This is because if the bias is strong, then cells will track the gradient very well. Consequently, they will move in nearly straight lines in the gradient direction, and straight movement corresponds to high persistence. This is a bias-induced persistence, distinct from the bias-independent persistence in the lower-right corner of the crescent. Finally, we see that the bending shape of the crescent implies that no solutions exist at large DP and intermediate CI (region III in Fig 6). In other words, it is not possible to have high persistence with partial bias. This is because, as mentioned above, persistence is induced either (i) directly, as a result of a large persistence parameter *p* which is independent of the bias, in which case the CI is low; or (ii) indirectly, as a result of a large bias parameter *b*, in which case the CI is high. Neither of these mechanisms permits intermediate bias, and therefore high persistence can be accompanied only by low or high directionality. Together, these features of the crescent shape imply that specific modes of chemotaxis are prohibited under our simple model, as indicated by the regions I, II, and III.

Finally, the crescent shape provides a qualitative rationale for the data from the simulations and experiments, which are overlaid in the cyan and red squares in Fig 6, respectively. Specifically, the shape of the crescent is such that if a cell has a low CI and intermediate DP (bottom right corner of the crescent) and its CI increases, its DP must decrease (solid magenta arrow in Fig 6). In contrast, a simultaneous increase in CI and DP from this starting position is not possible according to the model (dashed magenta arrow in Fig 6). We see that the data are qualitatively consistent with this predicted trend, as an increase in the CI corresponds to a decrease in the DP in both the experiments and the simulations (Fig 6, squares). There is quantitative disagreement, in the sense that the data do not quite overlap with the crescent, but this is a reflection of the extreme simplicity of the BPRW model. Nonetheless, the qualitative features of the BPRW model are sufficient to explain the way in which accuracy and persistence are mutually constrained during the chemotaxis response of these cells.

## Discussion

By integrating experiments with theory and simulations, we have investigated mutual constraints on the accuracy (CI), persistence (DP), and speed of cancer cell motion in response to a chemical attractant. We have found that while the CI of breast cancer cells increases with the strength of a TGF-*β* gradient, the speed does not show a strong trend, and the DP slightly decreases. The simulations suggest that the decrease in DP is due to a decoupling between sensing and persistence in the migration dynamics. The theory confirms that the decrease in DP is due to a mutual constraint on accuracy and persistence for this type of decoupled dynamics, and more generally, it suggests that entire regions of the accuracy–persistence space are prohibited.

The present results provide some insights into TGF-*β* induced migration mechanisms. Multiple signaling pathways induced by TGF-*β* affect the dynamics of actin polymerization regulating cell migratory behaviors [27, 43–45]. Among these, phosphatidylinositol 3-kinase (PI3K) and the small GTPase-Rac1 signaling have been reported to promote actin organization of breast cancer cells in response to TGF-*β* [45, 46]. PI3K and the Rho-family GTPase networks (including Rac1, RhoA and Cdc42) have been widely studied in chemotaxis, which regulates cell polarity and directional sensing [47–50]. The PI3K activity, thus, can possibly explain the present chemotactic responses of the breast cancer cells to TGF-*β* gradient. Recent studies have shown that PI3K is relevant to the accuracy of the cell movement in shallow chemoattractants, whereas it does not induce the orientation of cell movement in steep gradients; rather, PI3K contributes the motility enhancement [51, 52]. These results can be correlated with the cell motility trend in the present experimental results. In addition, the PI3K signaling pathway has been reported not to mediate the persistence of cell protrusions which could be directly related to the DP [47, 48]. The directional persistence could be more relevant to the polarity stability which is hardly controlled by chemotaxis [47] as presented in the present results. In TGF-*β* molecular cascades, activation of SMAD proteins could also affect the actin dynamics. Since SMAD-cascades include negative feedback inhibiting Rho activity [43, 44], it may affect the cell responses highly promoted in CI but not in speed. However, the underlying molecular mechanisms need further research.

Our finding that sensing and persistence are largely decoupled in the migration dynamics is related to the view that directional sensing and polarity are separate but connected modules in chemotaxis [11]. Indeed, CI, DP, and speed in our study play the roles of the directional sensing, polarity, and motility modules, respectively, that have been shown to reproduce many of the observed behaviors of chemotaxing cells. Moreover, several of the the molecular signaling pathways discussed above, including those involving PI3K and Rho family GTPases, have been proposed as the potential networks corresponding to these modules [11].

Several predictions arise from our work that would be interesting to test in future experiments. First, our simulation scheme assumes that the saturation of the CI with gradient strength (Fig 4A) is due to limited receptor numbers. However, alternative explanations exist that are independent of the receptors, such as the fact that it is more difficult to detect a concentration difference on top of a large concentration background than on top of a small concentration background due to intrinsic fluctuations in molecule number [30, 53]. An interesting consequence of our mechanism of receptor saturation is that, at very large gradients (beyond those of Fig 4A), the CI would actually decrease because all receptors would be bound. It would be interesting to test this prediction in future experiments.

Second, our work suggests that not all quadrants of the accuracy–persistence plane are possible for cells to achieve (Fig 6). It would be interesting to measure the CI and DP of other cell types, in other chemical or mechanical environments, to see if the crescent shape seen in Fig 6 is a universal restriction, or if not, what new features of chemotaxis are therefore not captured by the modeling. In this respect, the work here can be seen as a null model, deviations from which would indicate new and unique types of cell motion.

## Materials and methods

### Cell culture and reagents

Human breast adenocarcinoma cells (MDA-MB-231) were cultured in Dulbecco’s Modified Eagle Medium/Ham’s F-12 (Advanced DMEM/F-12, Lifetechnologies, CA, USA) supplemented by 5% v/v fetal bovin serum (FBS), 2 mM L-glutamine (L-glu), and 100 *μ*g ml^-1^ penicillin/streptomycin(P/S) for less than 15 passages. MDA-MB-231 cells were regularly harvested by 0.05% trypsin and 0.53mM EDTA (Lifetechnologies, CA, USA) when grown up to around 80% confluency in 75 cm^2^ T-flasks at 37 °C with 5% CO_2_ incubation. Harvested cells were used for experiments or sub-cultured.

Cell-matrix composition was prepared in the microfluidic device. For the composition, MDA-MB-231 cells were mixed with 2 mg/ml of type I collagen (Corning Inc., NY, USA) mixture prepared with 10X PBS, NaOH, HEPE solution, FBS, Glu, P/S, and cell-culture level distilled water after centrifuged with 1000 rpm for 3 minutes. The cell mixture was filled in center-channel of the microfluidic devices and incubated in at 37 °C with 5% CO_2_. The cells in the collagen matrix were initially cultured in basic medium (DMEM/F12 supplemented by 5% v/v FBS, 2 mM L-glu, and 100 *μ*g ml^−1^ p/s) for 24 hours. Then the cells were exposed by reduced serum medium for another 24 hours, which was advanced DMEM/F12 containing 1% v/v FBS, 2 mM L-glu, and 100 *μ*g ml^−1^ p/s [54]. After 24 hour-serum starvations, cells were exposed by a gradient of transforming growth factor beta-1 (TGF-*β*1, Invitrogen, CA, USA).

### Microfluidic device for chemical gradient

The microfluidic device was designed to generate a linear gradient of soluble factors (Fig 2). The device is composed of three channels which are 100 *μ*m in thickness as described previously [55]. A center channel that is 1 mm wide aims to culture tumor cells with ECM components. The center channel is connected to two side channels. The 300 *μ*m-wide side channels are connected to large reservoirs at the end ports including culture medium. Since the side channels are in contact with the top and bottom sides of the center channel, the growth factor gradient can be generated by diffusing the soluble factor from one of the side channels, a source channel, to the other, a sink channel. Assuming there is neither pressure difference nor flow between the side channels, the concentration of a given factor can be described by the chemical species conservation equation as follows:

$$\begin{array}{c} \frac{\partial c_{i}}{\partial t}=D_{i}\cdot \nabla c_{i} \end{array}$$ (13)

Once the concentration profile in the center channel reaches steady state, the linear profile persists for a while and can therefore be approximated by assuming the boundary conditions of concentration at the side channels are constants. To verify the diffusion behavior, the gradient formation was examined by using 10k Da FITC-fluorescence conjugated dextran (FITC-dextran). FITC-dextran solution was applied in the source channel while the sink channel was filled with normal culture medium. The FITC-dextran concentration profile was evaluated by the FITC fluorescent intensity in the center channel. To disregard the effect of photo-bleaching on the results, the intensity was normalized by the intensity of the source channel. The normalized intensity was reasonably considered since the fluorescence intensity of the source channel consistently remained as maximum due to the large reservoirs. The FITC dextran intensity profile (Fig 2C) showed that the linear profile was developed within 3 hours after applying the source and continued for more than 9 hours.

### Characterization of cell migration with time-lapse microscopy

Cell behaviors were captured every 15 minutes for 9 hours using an inverted microscope (Olympus IX71, Japan) equipped with a stage top incubator as described previously [56–58], so that the microfluidic platform could be maintained at 37 °C in a 5% CO_2_ environment during imaging. The time-lapse imaging was started 3 hours after applying TGF-*β*1 solution in the source channel to have sufficient adjusting time. To analyze each cell behavior, a cell area in the bright field images were defined by a contrast difference between the cells and a background, and the images were converted to monochrome images by using ImageJ. Cell trajectories were demonstrated by tracking centroids of the cell area. In tracking the cell movements, cells undergoing division were excluded to avoid extra influences to affect cell polarity [59]. Moreover, stationary cells due to the presence of the matrix were excluded [26, 59–61]. The stationary cells were defined as the cells that moved less than their diameter. A migration trajectory was defined by connecting the centroids of a cell from each time point.

### Statistical analysis of experiments

In examining the chemotactic characteristics of each group, more than 40 cell trajectories were evaluated per a group. A data point in Fig 3C–3E indicates each metric of a cell trajectory showing distribution characteristics with a box plot. The box plot includes boundaries as quadrants and a center as a median. The distribution of each metric was statistically analyzed by using Mann-Whitney U-test. This non-parametric method was used since the distribution was not consistently normal (the CI is a function of cosine). The significant change on the population lies on the biased distribution of each cell parameter when the *p* value < 0.05. Furthermore, the experiments were repeated at least 3 times and reported with means of medians ± standard estimated error (S.E.M.) in Fig 4A–4C. To evaluate physical limits on each metric, the data points were compared each other using a student t-test. The statistical significance between comparisons were examined when the *p* value < 0.05.

### Mathematical approximations

In the limit *p* = 0, Eqs 9 and 10 become

$$\begin{array}{ccc} \left\langle r^{2}\right\rangle & = & z^{2}M^{2}+2\left(1-2z^{2}\right)M+2\left(3z^{2}-1\right), \end{array}$$ (14)

$$\begin{array}{ccc} {\left\langle x\right\rangle }^{2} & = & z^{2}{\left(M-1\right)}^{2}, \end{array}$$ (15)

where *z* = *πb*, and we have neglected the exponential terms in the limit *M* ≫ 1. Defining the small parameter *ϵ* = 1/*M*, these expressions become

$$\begin{array}{ccc} \left\langle r^{2}\right\rangle & = & z^{2}M^{2}\left(1+c\epsilon \right), \end{array}$$ (16)

$$\begin{array}{ccc} {\left\langle x\right\rangle }^{2} & = & z^{2}M^{2}\left(1-2\epsilon \right) \end{array}$$ (17)

to first order in *ϵ*, where *c* ≡ 2(*z*^−2^ − 2). Inserting these expressions into Eqs 11 and 12, we obtain

$$\begin{array}{ccc} {\text{CI}}^{2} & = & 1-(c+2)\epsilon , \end{array}$$ (18)

$$\begin{array}{ccc} {\text{DP}}^{2} & = & z^{2}\left(1+c\epsilon \right) \end{array}$$ (19)

to first order in *ϵ*. Because *z* and *c* are both functions only of *b*, we eliminate *b* from Eqs 18 and 19 to obtain

$$\begin{array}{c} {\text{CI}}^{2}=1-2\epsilon \frac{1-{\text{DP}}^{2}}{{\text{DP}}^{2}} \end{array}$$ (20)

to first order in *ϵ*. This expression is equivalent to that given below Eq 12 and provides the left black line in Fig 6.

The right black line in Fig 6 corresponds to the maximal value of *p* for a given *b* that keeps Eq 8 non-negative. Non-negativity requires that the sum of the minimal values of each term in Eq 8 is zero: −*b* + *e*^−*p*^/[2*πI*_0_(*p*)] = 0. With this expression for *b* in terms of *p*, Eqs 11 and 12 become functions of only *p* and *M*. Therefore, by varying *p*, we compute the right black line parametrically.

## Supporting information

S1 FigComparison of directional persistence (DP) and directional autocorrelation time ($\tau_{\text{AC}}$).(A) Autocorrelation function for all trajectories in control experiment (no TGF-*β*); $\tau_{\text{AC}}$ is the integral under the curve. Plot of $\tau_{\text{AC}}$ vs. DP for control (gray), and 50 nM/mm TGF-*β* gradient condition (left blue triangle), as well as several other experimental conditions. Note that the relationship between $\tau_{\text{AC}}$ and DP is monotonic.(TIFF)

S2 Fig Cell trajectories for all values of TGF-*β* gradient strength, and all three experimental replicates. (TIF)

S1 Video Cellular Potts model (CPM) simulation. (GIF)

S1 Appendix Raw data of Figs 3 and 4. (XLSX)

## References

[1] Iglesias PA, Devreotes PN. Navigating through models of chemotaxis. Current Opinion in Cell Biology. 2008;20(1):35–40. 10.1016/j.ceb.2007.11.011 18207721

[2] Roussos ET, Condeelis JS, Patsialou A. Chemotaxis in cancer. Nature Reviews Cancer. 2011;11(8):573–587. 10.1038/nrc3078 21779009 PMC4030706

[3] Kim BJ, Hannanta-Anan P, Chau M, Kim YS, Swartz MA, Wu M. Cooperative roles of SDF-1*α* and EGF gradients on tumor cell migration revealed by a robust 3D microfluidic model. PloS ONE. 2013;8(7):e68422 10.1371/journal.pone.0068422 23869217 PMC3711811

[4] Varennes J, Mugler A. Sense and sensitivity: physical limits to multicellular sensing, migration, and drug response. Molecular Pharmaceutics. 2016;13(7):2224–2232. 10.1021/acs.molpharmaceut.5b00899 26835969

[5] Friedl P, Alexander S. Cancer invasion and the microenvironment: plasticity and reciprocity. Cell. 2011;147(5):992–1009. 10.1016/j.cell.2011.11.016 22118458

[6] Witsch E, Sela M, Yarden Y. Roles for growth factors in cancer progression. Physiology. 2010;25(2):85–101. 10.1152/physiol.00045.2009 20430953 PMC3062054

[7] Woodhouse EC, Chuaqui RF, Liotta LA. General mechanisms of metastasis. Cancer. 1997;80(S8):1529–1537. 9362419 10.1002/(sici)1097-0142(19971015)80:8+<1529::aid-cncr2>3.3.co;2-#

[8] Wang SJ, Saadi W, Lin F, Nguyen CMC, Jeon NL. Differential effects of EGF gradient profiles on MDA-MB-231 breast cancer cell chemotaxis. Experimental Cell Research. 2004;300(1):180–189. 10.1016/j.yexcr.2004.06.030 15383325

[9] Shields JD, Fleury ME, Yong C, Tomei AA, Randolph GJ, Swartz MA. Autologous chemotaxis as a mechanism of tumor cell homing to lymphatics via interstitial flow and autocrine CCR7 signaling. Cancer Cell. 2007;11(6):526–538. 10.1016/j.ccr.2007.04.020 17560334

[10] Petrie RJ, Doyle AD, Yamada KM. Random versus directionally persistent cell migration. Nature Reviews Molecular Cell Biology. 2009;10(8):538–549. 10.1038/nrm2729 19603038 PMC2752299

[11] Shi C, Huang CH, Devreotes PN, Iglesias PA. Interaction of motility, directional sensing, and polarity modules recreates the behaviors of chemotaxing cells. PLoS computational biology. 2013;9(7):e1003122 10.1371/journal.pcbi.1003122 23861660 PMC3701696

[12] Funamoto S, Milan K, Meili R, Firtel RA. Role of phosphatidylinositol 3 kinase and a downstream pleckstrin homology domain–containing protein in controlling chemotaxis in Dictyostelium. The Journal of Cell Biology. 2001;153(4):795–810. 10.1083/jcb.153.4.795 11352940 PMC2192389

[13] Mouneimne G, DesMarais V, Sidani M, Scemes E, Wang W, Song X, et al Spatial and temporal control of cofilin activity is required for directional sensing during chemotaxis. Current Biology. 2006;16(22):2193–2205. 10.1016/j.cub.2006.09.016 17113383

[14] Van Haastert PJ, Postma M. Biased random walk by stochastic fluctuations of chemoattractant-receptor interactions at the lower limit of detection. Biophysical Journal. 2007;93(5):1787–1796. 10.1529/biophysj.107.104356 17513372 PMC1948060

[15] Kay RR, Langridge P, Traynor D, Hoeller O. Changing directions in the study of chemotaxis. Nature Reviews Molecular Cell Biology. 2008;9(6):455–463. 10.1038/nrm2419 18500256

[16] Nelson RD, Quie PG, Simmons RL. Chemotaxis under agarose: a new and simple method for measuring chemotaxis and spontaneous migration of human polymorphonuclear leukocytes and monocytes. The Journal of Immunology. 1975;115(6):1650–1656. 1102606

[17] Iellem A, Mariani M, Lang R, Recalde H, Panina-Bordignon P, Sinigaglia F, et al Unique chemotactic response profile and specific expression of chemokine receptors CCR4 and CCR8 by CD4+ CD25+ regulatory T cells. The Journal of Experimental Medicine. 2001;194(6):847–854. 10.1084/jem.194.6.847 11560999 PMC2195967

[18] Mayr-Wohlfart U, Waltenberger J, Hausser H, Kessler S, Günther KP, Dehio C, et al Vascular endothelial growth factor stimulates chemotactic migration of primary human osteoblasts. Bone. 2002;30(3):472–477. 10.1016/S8756-3282(01)00690-1 11882460

[19] Fiedler J, Leucht F, Waltenberger J, Dehio C, Brenner RE. VEGF-A and PlGF-1 stimulate chemotactic migration of human mesenchymal progenitor cells. Biochemical and Biophysical Research Communications. 2005;334(2):561–568. 10.1016/j.bbrc.2005.06.116 16005848

[20] McCutcheon M. Chemotaxis in leukocytes. Physiological Reviews. 1946;26(3):319–336. 10.1152/physrev.1946.26.3.319 20993553

[21] Gorelik R, Gautreau A. Quantitative and unbiased analysis of directional persistence in cell migration. Nature Protocols. 2014;9(8):1931–1943. 10.1038/nprot.2014.131 25033209

[22] Codling EA, Plank MJ, Benhamou S. Random walk models in biology. Journal of the Royal Society Interface. 2008;5(25):813–834. 10.1098/rsif.2008.0014 PMC2504494 18426776

[23] Dang I, Gorelik R, Sousa-Blin C, Derivery E, Guérin C, Linkner J, et al Inhibitory signalling to the Arp2/3 complex steers cell migration. Nature. 2013;503(7475):281–284. 10.1038/nature12611 24132237

[24] Varennes J, Fancher S, Han B, Mugler A. Emergent versus individual-based multicellular chemotaxis. Physical Review Letters. 2017;119(18):188101 10.1103/PhysRevLett.119.188101 29219578

[25] Luwor RB, Hakmana D, Iaria J, Nheu TV, Simpson RJ, Zhu HJ. Single live cell TGF-*β* signalling imaging: breast cancer cell motility and migration is driven by sub-populations of cells with dynamic TGF-*β*-Smad3 activity. Molecular Cancer. 2015;14(1):50 10.1186/s12943-015-0309-1 25744371 PMC4343191

[26] Giampieri S, Manning C, Hooper S, Jones L, Hill CS, Sahai E. Localized and reversible TGF*β* signalling switches breast cancer cells from cohesive to single cell motility. Nature Cell Biology. 2009;11(11):1287 10.1038/ncb1973 19838175 PMC2773241

[27] Ikushima H, Miyazono K. TGF*β* signalling: a complex web in cancer progression. Nature Reviews Cancer. 2010;10(6):415 10.1038/nrc2853 20495575

[28] Kleuser B, Malek D, Gust R, Pertz HH, Potteck H. 17-*β*-Estradiol inhibits Transforming Growth Factor-*β* signalling and function in breast cancer cells via activation of Extracellular Signal-Regulated Kinase through the G protein coupled receptor 30. Molecular Pharmacology. 2008;74(6):1533–1543. 10.1124/mol.108.046854 18768737

[29] Venturoli D, Rippe B. Ficoll and dextran vs. globular proteins as probes for testing glomerular permselectivity: effects of molecular size, shape, charge, and deformability. American Journal of Physiology-Renal Physiology. 2005;288(4):F605–F613. 10.1152/ajprenal.00171.2004 15753324

[30] Ellison D, Mugler A, Brennan MD, Lee SH, Huebner RJ, Shamir ER, et al Cell–cell communication enhances the capacity of cell ensembles to sense shallow gradients during morphogenesis. Proceedings of the National Academy of Sciences. 2016;113(6):E679–E688. 10.1073/pnas.1516503113 PMC4760786 26792522

[31] Graner F, Glazier JA. Simulation of biological cell sorting using a two-dimensional extended Potts model. Physical Review Letters. 1992;69(13):2013 10.1103/PhysRevLett.69.2013 10046374

[32] Swat MH, Thomas GL, Belmonte JM, Shirinifard A, Hmeljak D, Glazier JA. Multi-scale modeling of tissues using CompuCell3D. Methods in Cell Biology. 2012;110:325 10.1016/B978-0-12-388403-9.00013-8 22482955 PMC3612985

[33] Szabó A, Ünnep R, Méhes E, Twal W, Argraves W, Cao Y, et al Collective cell motion in endothelial monolayers. Physical Biology. 2010;7(4):046007 10.1088/1478-3975/7/4/046007 21076204 PMC3044241

[34] Kabla AJ. Collective cell migration: leadership, invasion and segregation. Journal of The Royal Society Interface. 2012; p. rsif20120448. 10.1098/rsif.2012.0448 PMC3481577 22832363

[35] Varennes J, Han B, Mugler A. Collective chemotaxis through noisy multicellular gradient sensing. Biophysical Journal. 2016;111(3):640–649. 10.1016/j.bpj.2016.06.040 27508447 PMC4982970

[36] Frick CL, Yarka C, Nunns H, Goentoro L. Sensing relative signal in the Tgf-*β*/Smad pathway. Proceedings of the National Academy of Sciences. 2017; p. 201611428. 10.1073/pnas.1611428114 PMC5389321 28320972

[37] Shoval O, Goentoro L, Hart Y, Mayo A, Sontag E, Alon U. Fold-change detection and scalar symmetry of sensory input fields. Proceedings of the National Academy of Sciences. 2010; p. 201002352. 10.1073/pnas.1002352107 PMC2936624 20729472

[38] Wakefield LM, Smith DM, Masui T, Harris CC, Sporn MB. Distribution and modulation of the cellular receptor for transforming growth factor-beta. The Journal of Cell Biology. 1987;105(2):965–975. 10.1083/jcb.105.2.965 2887577 PMC2114751

[39] Mitchell E, Lee K, O’Connor-McCourt M. Characterization of transforming growth factor-beta (TGF-beta) receptors on BeWo choriocarcinoma cells including the identification of a novel 38-kDa TGF-beta binding glycoprotein. Molecular Biology of the Cell. 1992;3(11):1295–1307. 10.1091/mbc.3.11.1295 1333844 PMC275695

[40] Alt W. Biased random walk models for chemotaxis and related diffusion approximations. Journal of Mathematical Biology. 1980;9(2):147–177. 10.1007/BF00275919 7365332

[41] Othmer HG, Dunbar SR, Alt W. Models of dispersal in biological systems. Journal of Mathematical Biology. 1988;26(3):263–298. 10.1007/BF00277392 3411255

[42] Fraley SI, Feng Y, Giri A, Longmore GD, Wirtz D. Dimensional and temporal controls of three-dimensional cell migration by zyxin and binding partners. Nature Communications. 2012;3:719 10.1038/ncomms1711 22395610 PMC4380184

[43] Derynck R, Zhang YE. Smad-dependent and Smad-independent pathways in TGF*β* family signalling. Nature. 2003;425:577 10.1038/nature02006 14534577

[44] Moustakas A, Heldin CH. Dynamic control of TGF-*β* signaling and its links to the cytoskeleton. FEBS letters. 2008;582(14):2051–2065. 10.1016/j.febslet.2008.03.027 18375206

[45] Olson EN, Nordheim A. Linking actin dynamics and gene transcription to drive cellular motile functions. Nature Reviews Molecular Cell Biology. 2010;11(5):353–365. 10.1038/nrm2890 20414257 PMC3073350

[46] Dumont N, Bakin AV, Arteaga CL. Autocrine transforming growth factor-*β* signaling mediates Smad-independent motility in human cancer cells. Journal of Biological Chemistry. 2003;278(5):3275–3285. 10.1074/jbc.M204623200 12421823

[47] Krause M, Gautreau A. Steering cell migration: lamellipodium dynamics and the regulation of directional persistence. Nature reviews Molecular cell biology. 2014;15(9):577 10.1038/nrm3861 25145849

[48] Swaney KF, Huang CH, Devreotes PN. Eukaryotic chemotaxis: a network of signaling pathways controls motility, directional sensing, and polarity. Annual review of biophysics. 2010;39:265–289. 10.1146/annurev.biophys.093008.131228 20192768 PMC4364543

[49] Edlund S, Landstrom M, Heldin CH, Aspenstrom P. Transforming Growth Factor-*β*- induced Mobilization of Actin Cytoskeleton Requires Signaling by Small GTPases Cdc42 and RhoA. Molecular Biology of the Cell. 2002;13(3):902–914. 10.1091/mbc.01-08-0398 11907271 PMC99608

[50] Fukata M, Nakagawa M, Kaibuchi K. Roles of Rho-family GTPases in cell polarisation and directional migration. Current Opinion in Cell Biology. 2003;15(5):590–597. 10.1016/S0955-0674(03)00097-8 14519394

[51] Andrew N, Insall RH. Chemotaxis in shallow gradients is mediated independently of PtdIns 3-kinase by biased choices between random protrusions. Nature Cell Biology. 2007;9(2):193–200. 10.1038/ncb1536 17220879

[52] Bosgraaf L, Keizer-Gunnink I, Van Haastert PJ. PI3-kinase signaling contributes to orientation in shallow gradients and enhances speed in steep chemoattractant gradients. J Cell Sci. 2008;121(21):3589–3597. 10.1242/jcs.031781 18840645

[53] Mugler A, Levchenko A, Nemenman I. Limits to the precision of gradient sensing with spatial communication and temporal integration. Proceedings of the National Academy of Sciences. 2016;113(6):E689–E695. 10.1073/pnas.1509597112 PMC4760817 26792517

[54] Rhee S, Ho CH, Grinnell F. Promigratory and procontractile growth factor environments differentially regulate cell morphogenesis. Experimental Cell Research. 2010;316(2):232–244. 10.1016/j.yexcr.2009.09.021 19796636 PMC2787981

[55] Shin CS, Kwak B, Han B, Park K. Development of an in vitro 3D tumor model to study therapeutic efficiency of an anticancer drug. Molecular Pharmaceutics. 2013;10(6):2167–2175. 10.1021/mp300595a 23461341 PMC3880422

[56] Ozcelikkale A, Dutton JC, Grinnell F, Han B. Effects of dynamic matrix remodelling on en masse migration of fibroblasts on collagen matrices. Journal of The Royal Society Interface. 2017;14(135):20170287 10.1098/rsif.2017.0287 PMC5665820 28978745

[57] Ozcelikkale A, Shin K, Noe-Kim V, Elzey BD, Dong Z, Zhang JT, et al Differential response to doxorubicin in breast cancer subtypes simulated by a microfluidic tumor model. Journal of Controlled Release. 2017;266:129–139. 10.1016/j.jconrel.2017.09.024 28939108 PMC5723544

[58] Shin K, Klosterhoff BS, Han B. Characterization of Cell-Type-Specific Drug Transport and Resistance of Breast Cancers Using Tumor-Microenvironment-on-Chip. Molecular Pharmaceutics. 2016;13(7):2214–2223. 10.1021/acs.molpharmaceut.6b00131 27228477 PMC5032827

[59] Harley BAC, Kim HD, Zaman MH, Yannas IV, Lauffenburger DA, Gibson LJ. Microarchitecture of three-dimensional scaffolds influences cell migration behavior via junction interactions. Biophysical Journal. 2008;95(8):4013–4024. 10.1529/biophysj.107.122598 18621811 PMC2553126

[60] Clark AG, Vignjevic DM. Modes of cancer cell invasion and the role of the microenvironment. Current Opinion in Cell Biology. 2015;36:13–22. 10.1016/j.ceb.2015.06.004 26183445

[61] Haessler U, Teo JC, Foretay D, Renaud P, Swartz MA. Migration dynamics of breast cancer cells in a tunable 3D interstitial flow chamber. Integrative Biology. 2012;4(4):401–409. 10.1039/c1ib00128k 22143066

