# Supplementary figures and images for: Physical constraints on accuracy and persistence during breast cancer cell chemotaxis
Source: PLoS Comput Biol. 2019 Apr 10;15(4):e1006961. doi: 10.1371/journal.pcbi.1006961 (PMC6476516; doi:10.1371/journal.pcbi.1006961)

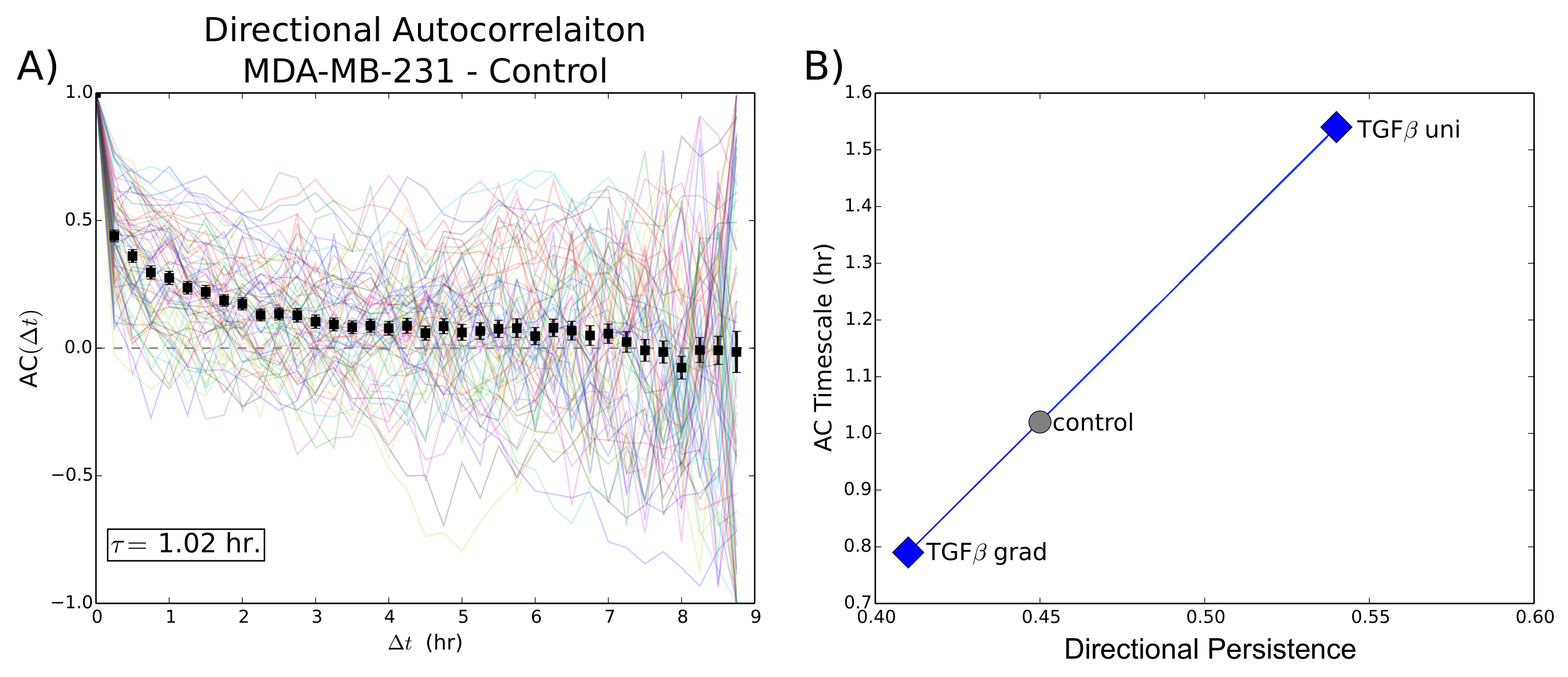

Supplement: S1 Fig — (A) Autocorrelation function for all trajectories in control experiment (no TGF-β); τAC is the integral under the curve. Plot of τAC vs. DP for control (gray), and 50 nM/mm TGF-β gradient condition (left blue triangle), as well as several other experimental conditions. Note that the relationship between τAC and DP is monotonic. (TIFF) [file pcbi.1006961.s001.tiff]

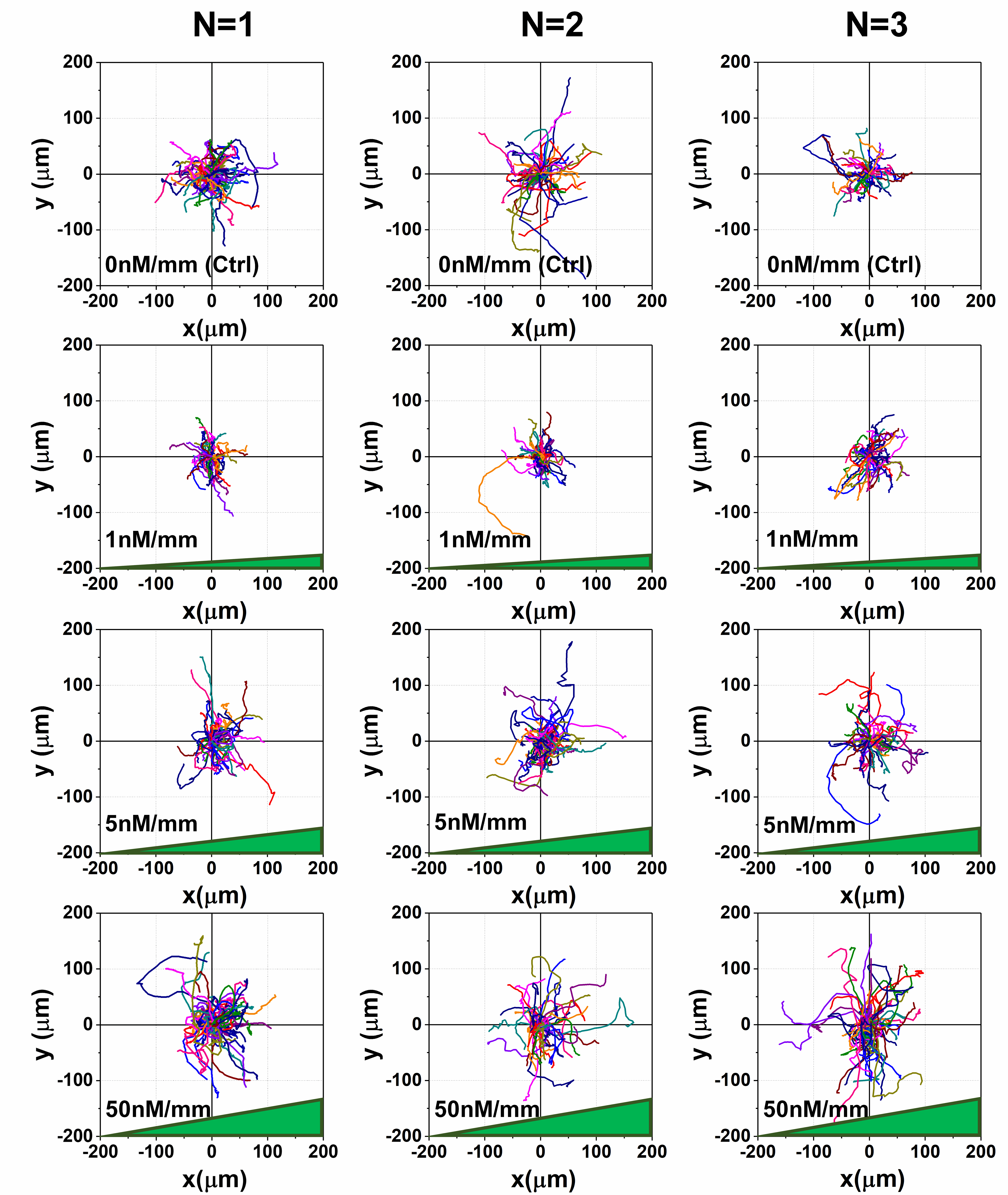

Supplement: S2 Fig — (TIF) [file pcbi.1006961.s002.tif]

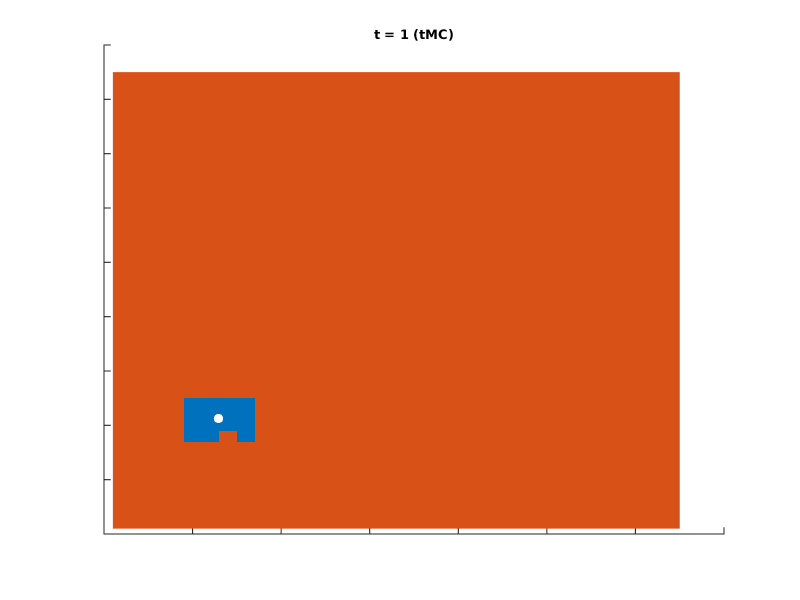

Supplement: S1 Video — (GIF) [file pcbi.1006961.s003.gif]
